# Time-Dependent Dynamics Required for the Degradation and Restoration of the Vascular Endothelial Glycocalyx Layer in Lipopolysaccharide-Treated Septic Mice

**DOI:** 10.3389/fcvm.2021.730298

**Published:** 2021-09-14

**Authors:** Akane Shinohara, Akira Ushiyama, Takehiko Iijima

**Affiliations:** ^1^Division of Anesthesiology, Department of Perioperative Medicine, Showa University, School of Dentistry, Tokyo, Japan; ^2^Department of Environmental Health, National Institute of Public Health, Saitama, Japan

**Keywords:** endothelial glycocalyx, lipopolysaccharide, dorsal skinfold chamber, bio-imaging, leukocytes-endothelium interaction

## Abstract

The endothelial glycocalyx (GCX) plays a key role in the development of organ failure following sepsis. Researchers have investigated GCX degradation caused by pathological conditions. Nonetheless, the GCX restoration process remains poorly understood. Herein, we developed a model in which GCX restoration could be reproduced in mice using *in vivo* imaging and a dorsal skinfold chamber (DSC). The severity of sepsis was controlled by adjusting the dose of lipopolysaccharide (LPS) used to trigger GCX degradation in BALB/c mice. We evaluated the GCX thickness, leukocyte-endothelial interactions, and vascular permeability using *in vivo* imaging through DSC under intravital microscopy. The plasma concentration of syndecan-1(Sdc-1), a GCX structural component, was also determined as a marker of GCX degradation. Thus, we developed a reproducible spontaneous GCX recovery model in mice. Degraded GCX was restored within 24 h by the direct visualization of the endothelial GCX thickness, and leukocyte-endothelial interactions. In contrast, indirectly related indicators of recovery from sepsis, such as body weight and blood pressure, required a longer recovery time. This model can be used to study intractable angiopathy following sepsis.

## Introduction

The endothelial glycocalyx (GCX) is important for endothelial function because it is involved in microvascular reactivity and modulates the interactions of the endothelium with blood constituents (1–7). GCX is composed of proteoglycans and glycosaminoglycans, produced by endothelial cell bodies. Its components must be periodically renewed, resulting in a turnover. However, the steps involved in the recovery of GCX remain unclear.

Several studies have reported that GCX gets degraded under pathological conditions, including sepsis and other diseases (8–13). Therefore, researchers have widely investigated the pharmacological interventions to preserve GCX, including the use of albumin (14), fresh frozen plasma (15, 16) and sevoflurane (17, 18) to establish therapeutic strategies for its restoration or protection. Unlike these biomaterials and chemicals, an additional approach to the restoration of GCX integrity relies on the intravascular treatment of GCX core components. The intravascular infusion of exogenous hyaluronan can partially prevent or completely reverse GCX damage induced by ischemia/reperfusion (I/R) (19). *In vivo*, the oral administration of sulodexide, a mixture of natural porcine heparan and dermatan sulfates, yield beneficial effects (20, 21). However, elucidating the natural GCX turnover and the theoretical protection and restoration of the GCX remains challenging. This can be attributed to the difficulties in observing the fragile and water-rich GCX structure.

Few studies have reported on the time required for GCX restoration after degradation. Giantsos-Adams et al. reported that GCX recovers in 12 h after enzymatic heparin sulfate degradation, upon stimulating endothelial cells by shear stress *in vivo* (22). In contrast, Potter et al. reported that 5–7 days are required for a restoration to its native hydrodynamically relevant thickness *in vivo*. Potter et al. used a digestive enzyme or cytokine for GCX degradation, and used fluorescent microparticle image velocimetry to quantify the GCX thickness (23). Two recent studies (24, 25) have focused on GCX restoration in the lung tissue. Yang et al. mentioned that fibroblast growth factor receptor 1/exostosin 1 signaling is necessary for GCX reconstitution, and that these homeostatic processes are impaired during sepsis. The reconstitution of the pulmonary endothelial surface layer (ESL), including the GCX, began 48 h after insult, and had nearly returned to the original thickness at 72 h following sepsis induction (24). Elucidating the mechanism of GCX reconstruction after its damage is extremely informative.

From a clinical perspective, the maintenance of GCX is important to avoid fluid overload, which contributes to postoperative fluid retention (26, 27). Endothelial damage because of GCX depletion causes hyperpermeability (28, 29). Leukocyte rolling on the surface of the endothelial lumen is an associated phenomenon that supposedly leads to hyperpermeability (30). However, the background mechanisms and temporal relationships responsible for the aforementioned phenomena remain unclear.

Herein, we aimed to develop an animal model for spontaneous GCX recovery, and to demonstrate the relationship between GCX recovery and various physiological parameters, including vitals and leukocyte adhesion.

## Materials and Methods

### Animal and Ethical Statement

All experiments were performed using 10- to 12-week-old male BALB/cCrSlc mice (Japan SLC Inc., Shizuoka, Japan), weighing 21 to 24 g. The mice were kept in an isolator rack (Super Mouse 1400TM Micro-Isolator Rack; Lab Products, Inc, Seaford, DE) in a 12 h light-dark cycle and under controlled temperature (23 ± 1°C) and humidity (50 ± 10%) conditions. Mice were fed a standard chow (FR-2, Funabashi Farm Co., Chiba, Japan) and water with free access. Before the experiments, mice were habituated in the animal facility for more than 1 week. All experimental protocols were approved by the Committee for Animal Experiments at the National Institute of Public Health (Protocol number 31-005) and were performed following all guidelines and laws for animal experiments in Japan. All animal experiments were in accordance with the Animal Research: Reporting of *in vivo* Experiments guidelines (31).

### Chemicals

Lipopolysaccharides (LPS) from *Escherichia coli* O26:B6, fluorescein isothiocyanate (FITC)-labeled wheat germ agglutinin (WGA) lectin from *Triticum vulgaris*, and tetramethylrhodamine (TMR)-labeled dextran (average molecular weight, 75 kDa [TMR-dex75]) were purchased from Sigma-Aldrich Co. (St Louis, MO). The mouse-soluble syndecan-1 (Sdc-1) enzyme-linked immunosorbent assay (ELISA) kit from Diaclone SAS (Besancon Cedex, France) was purchased. Ketamine hydrochloride, xylazine hydrochloride, and rhodamine 6G were purchased from Wako Pure Chemical Industries, Ltd. (Osaka, Japan). Sevoflurane was purchased from Pfizer Japan Inc. (Tokyo, Japan), and used for anesthesia using a small animal anesthetizer (MK-A110D, Muromachi Kikai Co., Ltd., Tokyo, Japan).

### LPS-Administered Septic Model and Overall Protocol of Animal Experiment

Sepsis was induced by intraperitoneal administration of LPS, as described in previous studies (25, 30). As the severity of the pathophysiological condition depends on the LPS dose, the dose was set to 2 mg/kg to allow the observation of spontaneous recovery following titration. LPS was administered intraperitoneally in two separate doses as follows: (i) an initial injection of 1 mg/kg and (ii) a second injection of 1 mg/kg administered 18 h later. Body weight was recorded daily for up to 10 days after LPS administration. Blood pressure was also measured daily up to 5 days by using a non-invasive blood pressure measuring device, which is based on the tail-cuff method (BP-98A-L; Softron Co, Tokyo, Japan). Measurements were performed between 10 am and 12 pm. Repetitive measurements were examined until three independent measurements were completed, and their mean value was employed as the blood pressure of the mouse on that day. Table 1 summarizes the overall protocol for the series of experiments.

**Table 1:** Overview of the experimental protocol.

	**0h**	**18h**	**24h**	**48h**	**72h**	**96h**	**120h**
LPS administration	↓	↓					
Body weight	∙		∙	∙	∙	∙	∙
Blood pressure	∙		∙	∙	∙	∙	-
Syndecan-1	∙		∙	∙	-	∙	-
Leukocyte adhesion	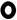		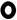	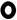	-	-	-
Thickness index of GCX	∙		∙	∙	∙	-	-
Permeability	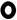		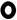	-	-	-	-

•*, measurement, 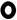, image acquisition. The experiments conducted at each time point are displayed. LPS was intraperitoneally administered to the mice immediately after the observation at 0 h. After 18 h, LPS was additionally administered. Moreover, a total dose of 2 mg/kg was administered. Measurements were performed at each time point, until the value recovered to the original value. ↓, LPS (1 mg/kg) administration*.

### Measuring Syndecan-1 Concentrations in Blood Plasma

The plasma concentration of Sdc-1 was used as a marker of GCX disruption. We collected a small volume of blood (approximately 200 μL) from the buccal venous plexus using an animal lancet (MEDIpoint, Inc. Mineola, NY) on each designated day. Plasma from the collected blood was frozen at −30°C. The plasma concentration was then quantified using the murine sCD138 (Sdc-1) ELISA Kit (Diaclone SAS, Besançon, France), according to the manufacturer's instructions.

### Dorsal Skinfold Chamber Preparation

A dorsal skinfold chamber (DSC) was surgically implanted into mice according to a previously reported method (32). The DSC was implanted at least 3 days before the scheduled observation to allow the animals to recover from acute inflammation caused by the surgical invasiveness of the procedure.

### Quantifying Leukocytes Interaction to the Endothelium

The adhesive leukocytes were counted over time using intravital microscopy. Leukocytes were fluorescently stained with rhodamine 6G (30, 32). Moreover, rhodamine 6G (100 μL) was administered intravenously to mice with implanted DSC. Each mouse was then set on the stage of a fluorescence microscope, and the leukocyte-endothelial interactions in both arterioles and venules were observed. Subjectively, a blood vessel with a diameter of 20–50 μm was selected, and a video for 15 s at three locations per mouse was recorded. The adhesive leucocytes within randomly selected regions of interest (ROIs) were manually counted for 15 s in a blinded fashion. Adhesiveness was classified into “adhering” and “rolling.” “Adhering” leukocytes was defined as leukocytes that do not move during the 15 s of measurement in the ROI. By contrast, “rolling” leukocytes were those that interacted with the endothelium and flowed more slowly than the blood. The adhering and rolling counts were calibrated according to a vascular diameter and length of 100 μm each.

To confirm the spatiotemporal relationship between the GCX layer and leukocyte adhesiveness, intravital double staining of GCX and leukocytes was performed with FITC-WGA lectin and rhodamine 6G, respectively. Fluorescent images were captured using appropriate fluorescent filters and fluorescence microscopy (BZ-X710, Keyence), and merged using an image analyzer optimized for the BZ-X710 microscope.

### Measuring GCX Thickness

The thickness of the vascular endothelial GCX was measured using *in vivo* imaging and stained with FITC-WGA lectin in DSC-implanted mice. Three mice were used for four time points because the toxicity of WGA-lectin might not be negligible following its repeated accumulation.

The mice were anesthetized with a cocktail of ketamine and xylazine, and FITC-WGA lectin was administered via the tail vein (6.24 mg/kg body mass). Images of arterioles and venules with a blood vessel diameter of 20–50 μm were captured using a fluorescent microscope (BZ-X710) with × 20 objective lens (SPlan Fluor ELWD, NA 0.45, Nikon Co., Tokyo, Japan). The images of seven arterioles and seven venules per mouse were randomly captured using an 8-bit gray scale and analyzed offline by using ImageJ software (National Institutes of Health, Bethesda, MD). To calculate the GCX thickness, we used a rectangular ROI (40 × 200 pixels) that included one side of the vascular endothelium. In the ROI, the long axis (200 pixels that corresponds to 75.4 μm) was adjusted to follow the direction of the blood flow (**Figure 4A**). The threshold value (*Th*) was calculated based on a brightness histogram of the ROI using the following formula:


$$\begin{array}{l} Th\;=\;F\left(I_{max}-I_{min}\right)+I_{min} \end{array}$$

where *I*_*max*_ is the maximum brightness value in the ROI, *I*_*min*_ is the minimum brightness value in the ROI, and *F* is an estimated value that allows an accurate capture of the GCX layer. In this study, *F* was set to 0.7 based on several trials.

Then, the ROI image was binarized according to the *Th* and inverted. The extracted black pixels were considered as the GCX layer. Thus, the thickness of the GCX could be measured if the total number of black pixels was divided by 200 pixels, and calibrated using a real scale. The obtained value was defined as the thickness index (TI) of the GCX.

### Measuring Vascular Permeability

TMR-dex75 was used to assess vascular permeability. A bolus (100 μL) of TMR-dex75 solution (3% [w/v] in saline) was administered via the tail vein to mice that had been anesthetized with sevoflurane. Three locations per mouse that included venules with a diameter of 20–50 μm in the DSC were randomly selected. Time-lapse images of these locations were saved every 30 min for up to 120 min using a fluorescent microscope (BZ-X710).

The vascular permeability area was confirmed in the three ROI squares (30 μm × 30 μm), adjacent to the blood vessel per series of the time-lapse images. The fluorescence intensity was measured inside each ROI, and it was subtracted from the control value. Changes in these values were defined as the vascular permeability index.

### Statistical Analyses

All data are presented as mean ± standard deviation. Data were statistically analyzed using SPSS Statistics software (version 23; Japan IBM Co., Tokyo, Japan). One-way analysis of variance (ANOVA) was used to compare the experimental data. Dunnett's test was performed for *post-hoc* comparisons. The statistical significance was set at *P* < 0.05.

## Results

### Physiological Outcomes

**Figure 1:**
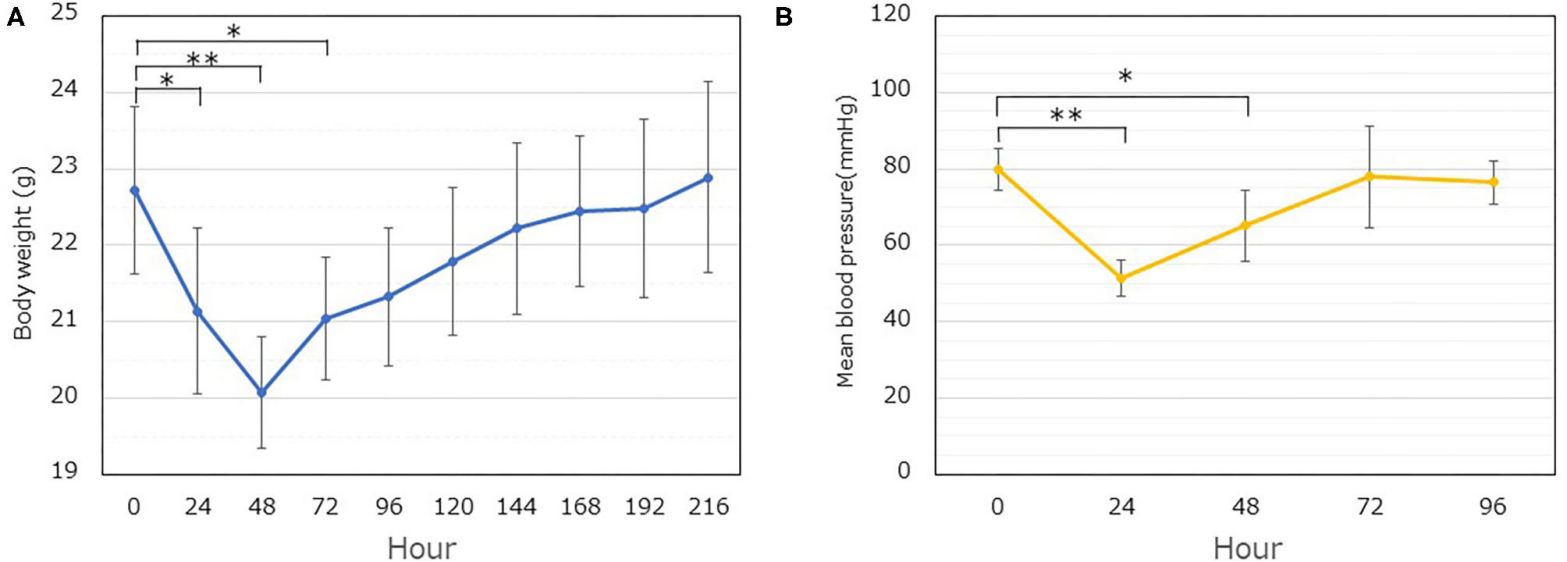
Time dependent changes in the body weight and mean blood pressure following LPS administration. **(A)** The body weight was followed for up to 216 h (9 days). The weight of the implanted dorsal skinfold chamber apparatus (1.5 g) was excluded. Significant differences are observed at 24, 48, and 72 h, compared to the value at 0 h (*P < 0.05, **P < 0.01). Each data item represents the mean ± SD (*n* = 8). **(B)** The blood pressure was measured. Each data item represents the mean ± SD of the mean blood pressure (*n* = 5) (*P < 0.05, **P < 0.01).

Table 1 summarizes the overall protocol for the series of experiments. The body weight and blood pressure reached a minimum at 48 h (Figure 1A, *P* < 0.01) and 24 h (Figure 1B, *P* < 0.01), respectively, following LPS administration. These significant decreases in the body weight and blood pressure were maintained for up to 72 h and 48 h, respectively. In addition, it took almost 168 h and 72 h to restore the body weight and blood pressure, respectively, to their original values (Figure 1).

### Measuring Syndecan-1 Concentrations in the Blood Plasma

**Figure 2:**
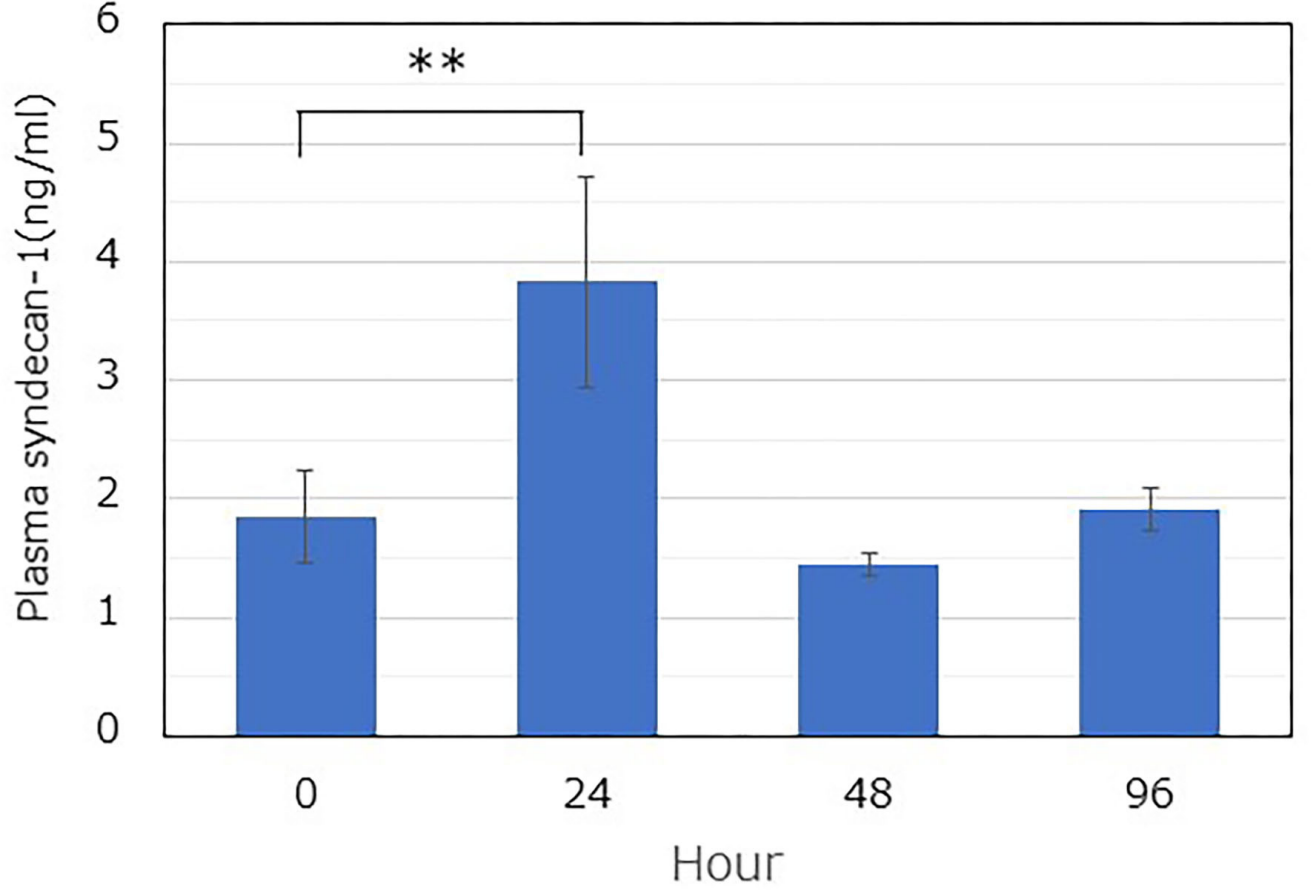
Time-dependent changes in the soluble plasma syndecan-1 level after LPS administration. The plasma level was determined using a commercially available ELISA kit. LPS was immediately administered after blood collection at 0 h. Blood was collected from similar mice (*n* = 3), and the plasma was extracted by centrifugation. A one-way ANOVA was performed, and the Dunnett's test was conducted for *post-hoc* comparisons (**P < 0.01).

The plasma Sdc-1 concentration, used as a marker of GCX degradation, was quantified using an ELISA kit. The concentration increased significantly at 24 h after administration, compared with the value at 0 h (1.85 ± 0.48 ng/mL [0 h] vs. 3.83 ± 1.09 ng/mL [24 h]) (Figure 2). However, it returned to the control value after 48 h.

### Quantifying Leukocyte-Endothelium Interaction

**Figure 3:**
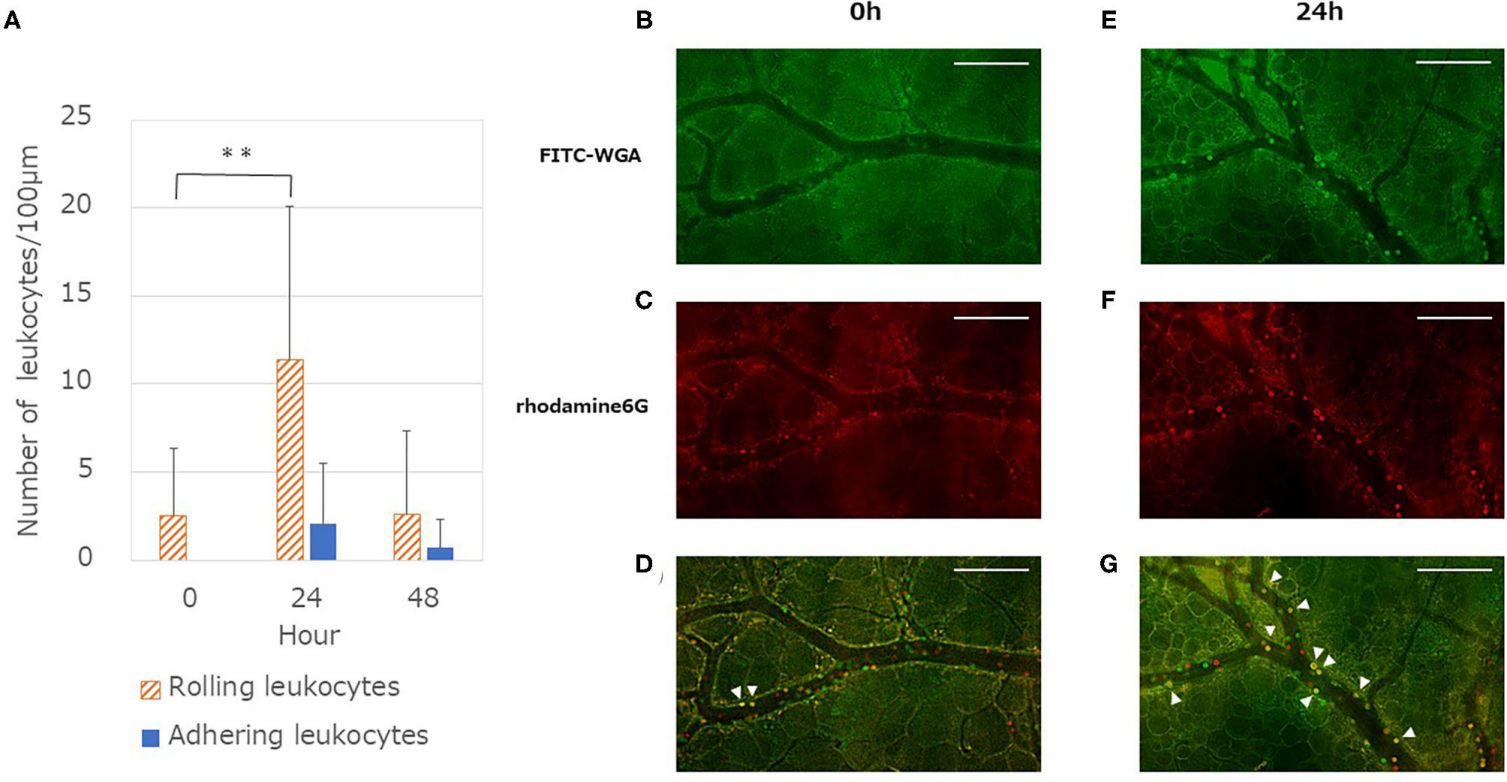
Quantitative analysis of leukocytes endothelium interactions after LPS administration. **(A)** Number of rolling leukocytes increased at 24 h and showed a significant difference compared with the values at 0 h [One-way ANOVA followed by Dunnett's test (**P < 0.01)]. By contrast, there is a small difference in the number of adhering leukocytes but this is not significant in the one-way ANOVA (*P* = 0.10). Each column represents the mean ± SD of 12 measurements from four mice at each time point. **(B,E)** Typical merged images of anesthetized mouse dorsal skin microvasculature: glycocalyx and leukocytes stained using FITC-WGA (green). **(C,F)** Typical merged images of microvasculature: cells containing mitochondria (leukocytes and platelets) stained using rhodamine 6G (red). Images in **(C,F)** captured 8 s after images in **(B,E)**. **(D)** Merged image of **(B,C)** captured at 0 h. **(G)** Merged image of **(E,F)** captured 24 h after LPS administration. Yellow cells (arrowheads) represent leukocytes that adhered to the endothelium. By contrast, red or green cells represent rolling leukocytes with weak interaction with the endothelium. Images modified (colored and merged) using commercial software. Scale bar: 100 μm.

The number of adhesive leukocytes (sum of rolling and adhering leukocytes) in the venules increased at 24 h vs. 0h (*P* < 0.01). Following 48 h, the number of adhesive leukocytes returned to the original level (Figure 3A). However, no significant difference was found in the arterioles.

The spatio-temporal relationship between the GCX and leukocyte adhesion is shown in Figure 3. The FITC-WGA lectin signal in the endothelium at 24 h was less intense than that at 0 h (Figures 3B,E). The number of leukocytes stained red by rhodamine 6G increased at 24 h, compared with that at 0h (Figures 3C,F). Yellow-stained leukocytes in merged images (Figures 3D,G) demonstrated that the leukocytes had tightly adhered to the endothelium, at least for the observation time (8 s). The time lag for capturing these two-color images enabled us to distinguish the tight adherence of leukocytes to the endothelium from those that were rolling/flowing along the endothelium.

### Measuring GCX Thickness

**Figure 4:**
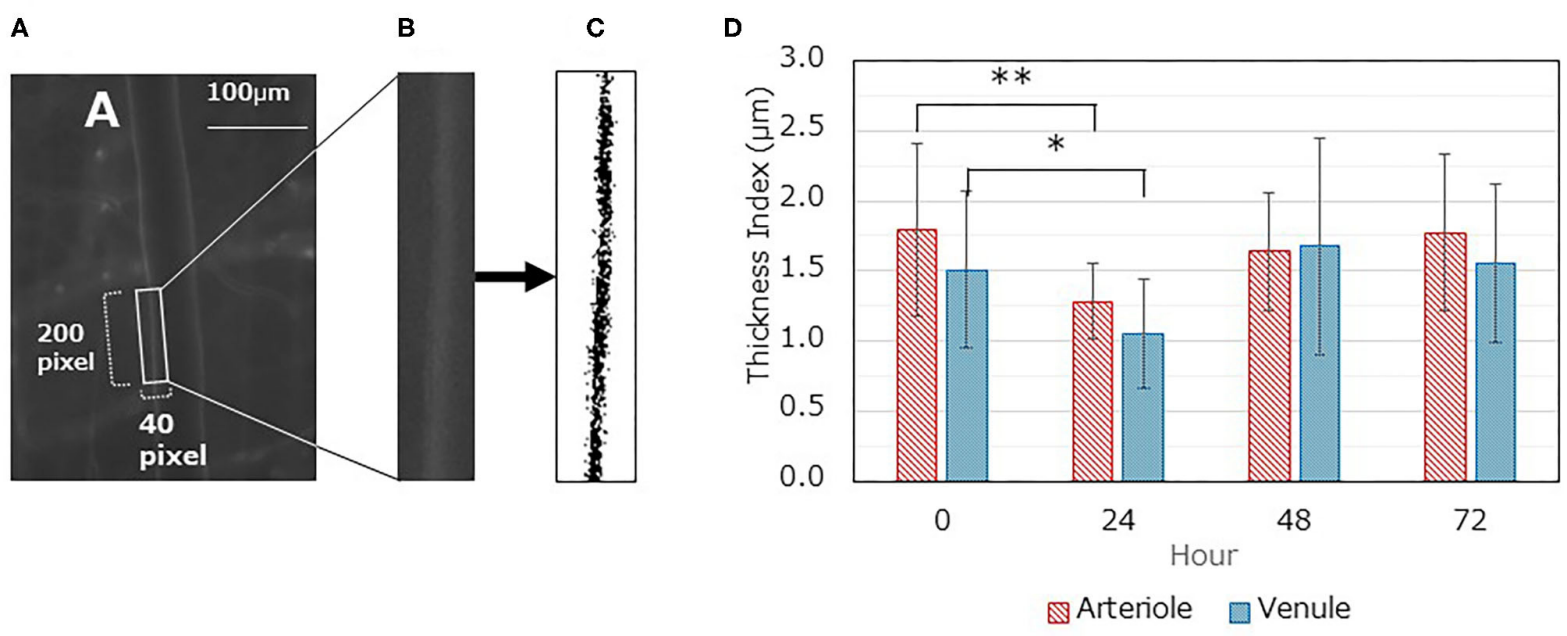
Changes in glycocalyx thickness index (TI) over time. **(A-C)** A typical image analysis procedure. **(A)** Gray image displaying fluorescent FITC-WGA lectin; the region of interest (ROI) (40 × 200 pixels) is set as containing one side of the vascular endothelium. The long axis is set similar to the blood flow direction. **(B)** The ROI image was saved as a separate file. **(C)** The saved ROI image is binarized according to the threshold value and inversed. The TI value was then calculated. **(D)** Time dependent changes in TI for both arterioles and venules after LPS administration. Each data item represents the mean ± SD for 21 measurements from three mice for each condition. A one-way ANOVA was performed, and Dunnett's test was conducted for *post-hoc* comparisons (*P < 0.05, **P < 0.01).

The GCX in the microvasculature was fluorescently labeled with FITC-WGA lectin, and the thickness of the fluorescent layer was quantified using image analysis. The thickness of the GCX decreased in both arterioles and venules at 24 h after LPS administration [arteriole, 1.28 ± 0.27 (control) vs. 1.79 ± 0.62, *P* < 0.01; venule, 1.05 ± 0.39 vs. 1.51 ± 0.56, *P* < 0.05] (Figure 4D). However, the thickness recovered 48 h after LPS administration (vs. control, *P* > 0.05).

### Measuring Vascular Permeability

**Figure 5:**
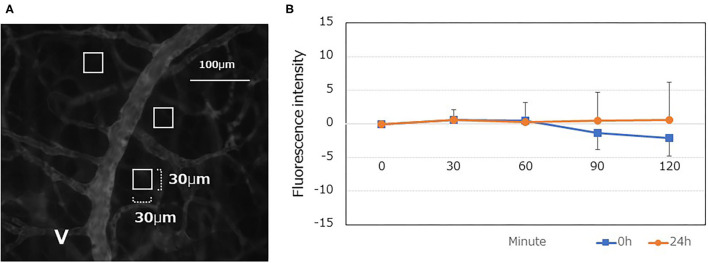
Quantitative analyses of the vascular permeability of TMR-dex75 at 0, and 24 h after LPS administration. **(A)** Typical fluorescent gray image following TMR-75 administration (0 min). Interstitial tissue containing as few capillary blood vessels as possible was randomly selected, and a square region of interest (ROI) was set at three locations (30 × 30μm each). The mean fluorescence intensity within the ROI was calculated using ImageJ. The ROI was followed up at 30, 60, 90, and 120 min to determine the fluorescence intensity of the interstitial tissue. **(B)** A time-dependent increase was not observed even at 24 h after LPS administration (*n* = 9).

A total of 9 mice were used, of which 3 were used for the analysis at 0 h and 6 were for 24 h. In the experimental procedure, three microvascular images inside the DSC were saved per mouse. In the analysis, three squared ROIs were set for each of three saved images locations. Therefore, 27 measurements at 0h and 54 measurements at 24h were examined respectively. Despite an extravascular leakage of TMR-dex75 observed for up to 120 min, no significant increase was found (Figure 5).

## Discussion

In this study, we monitored the recovery of degraded GCX. A certain level of recovery was achieved within 24 h, following the peak of GCX degradation in a septic mouse model with DSC implants.

In our previous report, GCX was degraded by LPS treatment, resulting in increased leukocyte-endothelial interactions and vascular permeability (30). In the present study, we attempted to elucidate the spatiotemporal restoration of GCX post-degradation. As an index of GCX degradation, we quantified the GCX thickness based on fluorescent images and FITC-WGA lectin staining. To validate the TI, we quantified the plasma Sdc-1 concentration, a marker of GCX degradation (33, 34). Furthermore, we analyzed the daily changes in body weight and mean blood pressure as systemic outcomes to determine the recovery from the pathophysiological condition. The decrease in GCX TI and the increase in the plasma Sdc-1 concentration plateaued at 24 h after LPS administration. In addition, these parameters returned to their original levels at 48 h. Moreover, the blood pressure reached its lowest value at 24 h, and returned to the original level at 72 h. The body weight reached its lowest value at 48 h, and required ≥168 h to return to its original level. Thus, a recovery from the impact of septic insult took longer than the recovery of the GCX structure, implying that GCX restoration occurs relatively rapidly, and preceded the restoration of physical conditions in the body.

One of the important physiological roles of the GCX is vascular protection via the inhibition of leucocyte adhesion. Under the pathophysiological condition, the GCX is targeted and shed by inflammatory mediators. LPS administration increases the production of cytokines such as tumor necrosis factor-α, interleukin-6, and interleukin-8 from immune cells. These activate matrix metalloproteinases and other degrading enzymes that degrade the components of GCX. This results in the fragmented release of Sdc-1 into the blood (35). Along with the unveiling of the GCX, adhesion molecules are exposed and facilitate ligand–receptor interactions that promote the adhesion of leukocytes (28, 36, 37). From this point of view, the measurement of real time leukocyte-endothelial interaction using intravital microscopy by applying a dorsal skinfold chamber is considered very important (30, 38). Our findings suggested that both leukocyte-endothelial interactions and plasma Sdc-1 concentrations were inversely correlated with the GCX TI. This is probably the first report on the relationship between GCX integrity and leukocyte behavior. In other words, the restoration of GCX regulated adhesion-related molecules located on the endothelial surface, such as selectins, ICAM-1 and VCAM-1, and suppressed inflammatory response.

Few studies have reported on the restoration of GCX after degradation. It takes several days to restore the GCX (22–25). Despite similar results, a direct comparison is difficult because of the differences in the experimental procedures and target organs. Inagawa et al. used a septic mouse model with LPS injection (25). They reported that plasma Sdc-1 levels peaked at 24 h, and returned to baseline at 48 h following LPS injection. This does not mean the restoration of the GCX, but may suggest that the GCX degradation process is interrupted. Moreover, GCX restoration following LPS injection required 72–96 h to return to the control level by observation with electron microscopy. Some of these findings were similar to ours (Figures 2, 4). However, the time required for restoring GCX thickness to the control level was longer than the present results. This difference could be attributed to several possible reasons as follows: (i) differences in the technique used to identify GCX (electron microscopy vs. fluorescent intravital microscopy) and (ii) differences in the severity of induced sepsis. Inagawa et al. reported on a survival rate of 21% at 48 h in LPS-treated mice, compared with a rate of 100% at 48 h in our experiment. The severity of induced sepsis reportedly depends on both the amount of LPS administered and the mouse strain (25).

Notably, our study failed to confirm hyperpermeability following sepsis. Our previous study (30) reported on the time-dependent permeability of TMR-dex75. This difference can be attributed to the impact of induced sepsis. We used an LPS dose of 4 mg/kg in the previous study, resulting in a 48% survival rate (30), compared with a dose of 2 mg/kg in the present study, resulting in a 100% survival rate. Considering our aim to clarify the GCX recovery process, we chose not to use a model with a low survival rate. These differences can be explained by the hypothesized mechanism called the “double barrier concept” (39). This concept is based on the idea that if only the GCX disruption occurs, an intact second barrier, “tight junctions” remains in the region of the vasculature. The tight junctions restrict the passage of macromolecules, and when an injury affects both the GCX and the tight junctions, the permeability of the vessel wall is thought to increase significantly. Recently, we reported a similar phenomenon that the structural barrier of the GCX does not solely determine the permeability of the endothelial layer, since enzymatic depletion of the GCX did not increase the permeability (40). Therefore, the simple digestion of the GCX cannot induce hyperpermeability, owing to the failure of the opening signal required for loosening of the endothelial tight junction to enter. Our model demonstrated that leukocyte-endothelium interactions occur after GCX damage. The role of leukocytes in the release of the opening signal for tight junctions should increase the permeability. This necessitates exploring the mechanism of hyperpermeability. Considering the above-mentioned putative mechanism, researchers might need to examine an animal model with vascular hyperpermeability under sepsis for promoting the research on GCX restoration.

Herein, we presented the time-dependent dynamics required for the degradation and restoration of vascular endothelial GCX. GCX can be rapidly restored, and precedes systemic recovery. In conclusion, our findings improve our understanding of the recovery of degraded GCX. To our knowledge, this is the first study to document the time required for GCX restoration under septic conditions, using intravital microscopy. Our findings provide important basic information for exploring strategies to protect or restore GCX.

## Data Availability Statement

The original contributions presented in the study are included in the article/supplementary material, further inquiries can be directed to the corresponding author/s.

## Ethics Statement

The animal study was reviewed and approved by Committee for Animal Experiments at the National Institute of Public Health, Japan.

## Author Contributions

AS, AU, and TI wrote the manuscript. AS performed all the experiments. AU supervised the animal study. TI edited the manuscript. All authors contributed to the article and approved the submitted version.

## Funding

This study was supported by the Japan Society of the Promotion of Science (JSPS) KAKENHI Grant-in-Aid for Scientific Research (C) 16K11762 to TI.

## Conflict of Interest

The authors declare that the research was conducted in the absence of any commercial or financial relationships that could be construed as a potential conflict of interest.

## Publisher's Note

All claims expressed in this article are solely those of the authors and do not necessarily represent those of their affiliated organizations, or those of the publisher, the editors and the reviewers. Any product that may be evaluated in this article, or claim that may be made by its manufacturer, is not guaranteed or endorsed by the publisher.
